# Decreased Expression of NUSAP1 Predicts Poor Overall Survival in Cervical Cancer

**DOI:** 10.7150/jca.34640

**Published:** 2020-02-21

**Authors:** Qiqi Xie, Wen Ou-yang, Mingwei Zhang, Huimei Wang, Qiuyuan Yue

**Affiliations:** 1 Department of Radiology, Fujian Cancer Hospital & Fujian Medical University Cancer Hospital, Fuzhou, Fujian 350014, People's Republic of China; 2 Department of Radiation Oncology, First Affiliated Hospital of Fujian Medical University Chazhong Road No. 20, Fuzhou, Fujian 350005, People's Republic of China; 3 Department of Orthopaedics, Second Hospital of Lanzhou University, Lanzhou, Gansu, 730030, People's Republic of China; 4 The Second Clinical Medical College, Zhujiang Hospital, Southern Medical University, Guangzhou 510282, People's Republic of China; 5 Department of Integrative Medicine and Neurobiology, State Key Laboratory of Medical Neurobiology, Institute of Brain Science, School of Basic Medical Sciences, Shanghai Medical College, Fudan University, Shanghai, 200032, People's Republic of China; 6 Institute of Immunotherapy, Fujian Medical University, Fuzhou, Fujian 350122, People's Republic of China; 7 Fujian Medical University Union Hospital, Fuzhou, Fujian 350004, People's Republic of China; 8 Morning Star Academic Cooperation, Shanghai

**Keywords:** NUSAP1, CESC, prognosis, biomarker, TCGA, GEO

## Abstract

**Background:** Nucleolar and spindle-associated protein 1 (NUSAP1) was previously reported to be associated with poor prognosis in multiple cancers. In the present study, we comprehensively investigated the clinicopathological features and potential prognostic value of NUSAP1 in cervical squamous cell carcinoma and endocervical adenocarcinoma (CESC).

**Methods:** The expression profiles of the genes were extracted from Gene Expression Omnibus (GEO), The Cancer Genome Atlas (TCGA), International Cancer Genome Consortium (ICGC), Cancer Cell Line Encyclopedia (CCLE), Gene Expression Profiling Interactive Analysis (GEPIA), and The Human Protein Atlas databases. The association between clinicopathological characteristics and NUSAP1 was analyzed using logistic regression in TCGA patients and receiver operating characteristic (ROC) curve analysis for GSE7803, GSE9750, and GSE63514 datasets. The prognostic value of NUSAP1 in TCGA patients was evaluated using the Kaplan-Meier method and Cox regression. Gene set enrichment analysis (GSEA) was conducted using TCGA dataset.

**Results:** A total of 68 differentially expressed genes (DEGs) were identified in CESC. ROC analysis of NUSAP1 suggested that the area under the ROC curve was 0.968. Kaplan-Meier survival analysis indicated that CESC with low expression of NUSAP1 has a worse prognosis than CESC with high NUSAP1 expression (P = 0.005). The logistic regression revealed that low NUSAP1 expression in CESC was related to advanced tumor stage in TCGA database. Moreover, Cox regression analysis showed that NUSAP1 expression correlated significantly with prognosis in the case of patients in TCGA database. GSEA demonstrated that CESC patients with high expression of NUSAP1 were enriched in the G2M checkpoint, MYC targets, and breast cancer ZNF217.

**Conclusion:** The results suggest that identification of DEGs might enhance our understanding of the causes and molecular mechanisms underlying the development of CESC. Moreover, NUSAP1 may play an important role in CESC progression and prognosis and may serve as a valuable indicator of poor survival in CESC.

## Introduction

Cervical cancer is the third most commonly diagnosed carcinoma and the fourth most prevalent cause of cancer-associated mortality in women, worldwide, with 530,000 new cases and 275,000 deaths reported every year 1. In clinical practice, patients with early stage cervical cancer (stages I to IIA) are mainly treated with surgery, whereas those with stage IIB to IV cancer are treated with chemoradiotherapy 2. However, the rate of recurrence of cancer is approximately 20%-25%, and the 5-year survival rate for advanced stage cervical cancer remains poor, being less than 50% 3. The International Federation of Gynecology and Obstetrics (FIGO) stage system based on anatomical characteristics has been demonstrated to be one of the most important prognostic factors in determining the therapeutic protocols. However, significant differences in survival are reported in the same FIGO staging, suggesting that more prognostic markers are needed for reflecting the biodiversity of cancer, improving patient risk stratification, and for investigating individual therapeutic schemes.

The dysregulation of mitosis is appreciated as a hallmark of malignant tumors. Nucleolar and spindle-associated protein 1 (NUSAP1) is an essential microtubule and chromatin-binding protein, which stabilizes and cross-links microtubules during mitosis 4, manages chromosome oscillation, and regulates the dynamics of kinetochore microtubules. Numerous evidences have revealed a critical role for NUSAP1 in mitosis 5, 6. In addition, NUSAP1 has been demonstrated to be involved in the development and prognosis of several cancers. It was found that NUSAP1 is overexpressed in hepatocellular cancer tissues when compared with noncancerous liver tissue 7. Although the exact molecular mechanisms underlying the effect of NUSAP1 warrant further confirmation, the observations highlight that NUSAP1 may serve as an attractively prognostic marker for cancer progression and metastasis, and may provide new insights for decreasing the risk of recurrence and morbidity.

Results of gene expression profiling of NUSAP1 suggest that it may play a critical role in cervical cancer 8. Moreover, the diagnostic sensitivity and specificity of NUSAP1 were around 90%. However, the association between NUSAP1 expression and prognosis and its biological functions and correlation with other predominant clinical factors of cervical cancer have not been investigated. Bioinformatic analysis based on multiple Gene Expression Omnibus (GEO, http://www.ncbi.nlm.nih.gov/geo) 9 datasets has permitted the mapping of gene expression signature and exploration of the differential expression of NUSAP1 in the development of cervical cancer 10. Additionally, Gene Set Enrichment Analysis (GSEA) revealed the common biological pathways by focusing on gene sets to interpret gene expression data 11. Furthermore, the extraction of the patient follow-up data from the Cancer Genome Atlas (TCGA) database might help in exploring the prognostic value of NUSAP1 in cervical cancer and its correlations with other major clinical factors.

In the present study, differentially expressed genes (DEGs) were identified using the data present in the GEO, TCGA, and International Cancer Genome Consortium (ICGC) databases. Subsequently, the diagnostic and prognostic values of NUSAP1 in carcinoma and endocervical adenocarcinoma (CESC) were determined.

## Materials and Methods

### Datasets

The microarray data from three gene expression profile datasets (GSE7803, GSE9750, and GSE63514) were downloaded from the GEO database. Both GSE7803 and GSE9750 were obtained from the GPL96 platform [HG-U133A] Affymetrix Human Genome U133A Array. GSE7803 includes data from 21 CESC samples and 10 normal samples, whereas GSE9750 includes data from 33 CESC samples and 24 normal samples. GSE63514 was obtained from the GPL570 platform [HG-U133_Plus_2] Affymetrix Human Genome U133 Plus 2.0 Array and includes data from 104 CESC samples and 24 normal samples. RNA-sequencing gene expression data from TCGA were retrieved on September 27, 2018, and comprised data from 306 cervical cancer samples and 3 normal samples. The ICGC data included gene-expression data from cervical squamous cell carcinoma projects (data released on April 30, 2018).

### Identification of differentially expressed genes (DEGs) and gene function analysis

The Affy 12 and limma packages 13 in Bioconductor (v1.46.1) were applied on microarray data to filter the DEGs between the CESC patient group and the normal group. Background correction, normalization, removal of batch effect, and calculation of expression were included in the procedures. The P values of DEGs were analyzed using Student's *t*-test performed using the limma package. TCGA data were analyzed using the edgR package, whereas because the ICGC does not provides the original sample information, the difference analysis was done online. The cut-off threshold in GEO was |log fold change (FC)| ≥ 1 and adjusted P value < 0.05 and those in TCGA and ICGC analysis were adjusted P value < 0.01 and |log fold change (FC)| ≥ 2. Moreover, the Gene Ontology Consortium was used to provide a comprehensive, dynamic, and controlled source for functional genomics 1, including molecular function (MF), biological process (BP), and cellular component (CC). The gene ontology (GO) enrichment analysis was conducted using the DAVID database. Subsequently, the GOplot R package was used for drawing the bubble plot.

### Integration of protein-protein interaction (PPI) network

The cBio Cancer Genomics Portal (cBioPortal) is an open-access Web platform for exploring, visualizing, and analyzing multidimensional cancer genomics data 14. NetworkAnalyst 15 serves as a comprehensive web-based tool to conduct various meta-analyses of gene expression data. The Cytoscape software 16 was applied to map the protein-protein interaction (PPI) network and to analyze the interaction among candidate DEGs encoding proteins in CESC. First, the online database, cBioPortal, was utilized to build a co-expression network and to determine the DEGs encoding proteins in CESC. Second, the NetworkAnalyst was used to construct a PPI network (confidence score = 900). Third, the PPI network was input in the Cytoscape software and the NetworkAnalyst plug-in was utilized to calculate the node degree and for visualization.

### Gene set enrichment analysis (GSEA)

GSEA is a statistical method to assess whether apriori defined set of genes shows statistically significant, concordant differences between two different biological status 17. In the present study, GSEA generated an ordered list of all genes and a single gene, NUSAP1, in CESC. GSEA was conducted to demonstrate significant differences in survival between CESC and normal patients for all the genes and between high and low NUSAP1 expression groups to identify the signaling pathways regulated by CESC-related genes and NUSPA1 using GEO matrix of GSE7803 and GSE9750 as raw data after removing the batch effect. A phenotype label was applied for the expression level of all the genes and NUSAP1. The normalized enrichment score (NES) and the nominal P value were used for calculating and sorting the enriched pathways in each phenotype. C2.all.v6.2.symbols.gmt was selected as the reference gene set. The gene set permutations were performed 1000 times.

### Survival analysis

The gene expression data (306 cases) and relevant clinical information for the CESC patients were downloaded from TCGA website for further analyses. All statistical analyses were performed using R. The relationship between clinicopathologic characteristics and NUSAP1 were analyzed with the logistic regression and receiver operating characteristic (ROC) methods. The clinicopathological features related to the overall survival of CESC patients in TCGA database were determined using the Kaplan-Meier method and Cox regression analysis. The cut-off value of NUSAP1 expression was based on the best separation.

The diagnostic ROC curve was used to explore the prognostic or predictive accuracy of each characteristic underlying the area under curve (AUC). The “pROC” of R package was used to perform the ROC curve analysis. In the present study, we screened the GEO datasets (GSE7803, GSE9750, and GSE63514).

The Kaplan-Meier curve was used to estimate the effects of NUSAP1 on the overall survival of CESC patients. Based on the best separation, the patients were classified into NUSAP1-low and NUSAP1-high expression groups. The difference between low or high NUSAP1 expression groups was assessed by log-rank test with R package “survival”. A P value less than 0.05 was identified as significant.

### Expression analysis of NUSAP1

To further explore the clinical value of NUSAP1, matrix plots were used to display the expression patterns of NUSAP1. Scatterplots for NUSAP1 expression were obtained from Broad Institute Cancer Cell Line Encyclopedia (CCLE), The Human Protein Atlas, and Gene Expression Profiling Interactive Analysis (GEPIA, http://gepia.cancer-pku.cn/). The Human Protein Atlas is an open access database mapping all the human proteins in organs, tissues, and cells and uses integration of various omics technologies 18. CCLE is a collection of RNA-seq datasets, including whole genome and whole exome, encompassing nearly 1000 human cancer cell lines and provides public access for analysis and visualization 19. GEPIA is a web server supporting normal and cancer gene expression profiling and interactive analyses 20. The mRNA and protein expression of NUSAP1 in organs, tissues, and cell lines were verified by CCLE, The Human Protein Atlas, and GEPIA.

### Statistical analysis

All statistical analyses were performed using R. Logistic regression analysis was used to identify the relationship between clinicopathologic characteristics and the expression of NUSAP1. Clinicopathologic characteristics were related to the overall survival in CESC patients using the Kaplan-Meier method and Cox regression analysis. The Cox regression model was used to conduct univariate and multivariable survival analyses. Multivariate Cox analysis was conducted to compare the influence of NUSAP1 expression on survival along with other characteristics. Variables with P value ≤ 0.05, including hysterectomy performed, stage, status, distant metastasis, and locoregional recurrence, were entered in the multivariate Cox regression analysis as categorical variables. The cut-off value of NUSAP1 expression was set, based on the best separation. Statistical significance for a two-tailed test was set at 0.05.

## Results

### Selection and functional analysis of DEGs

**Figure 1:**
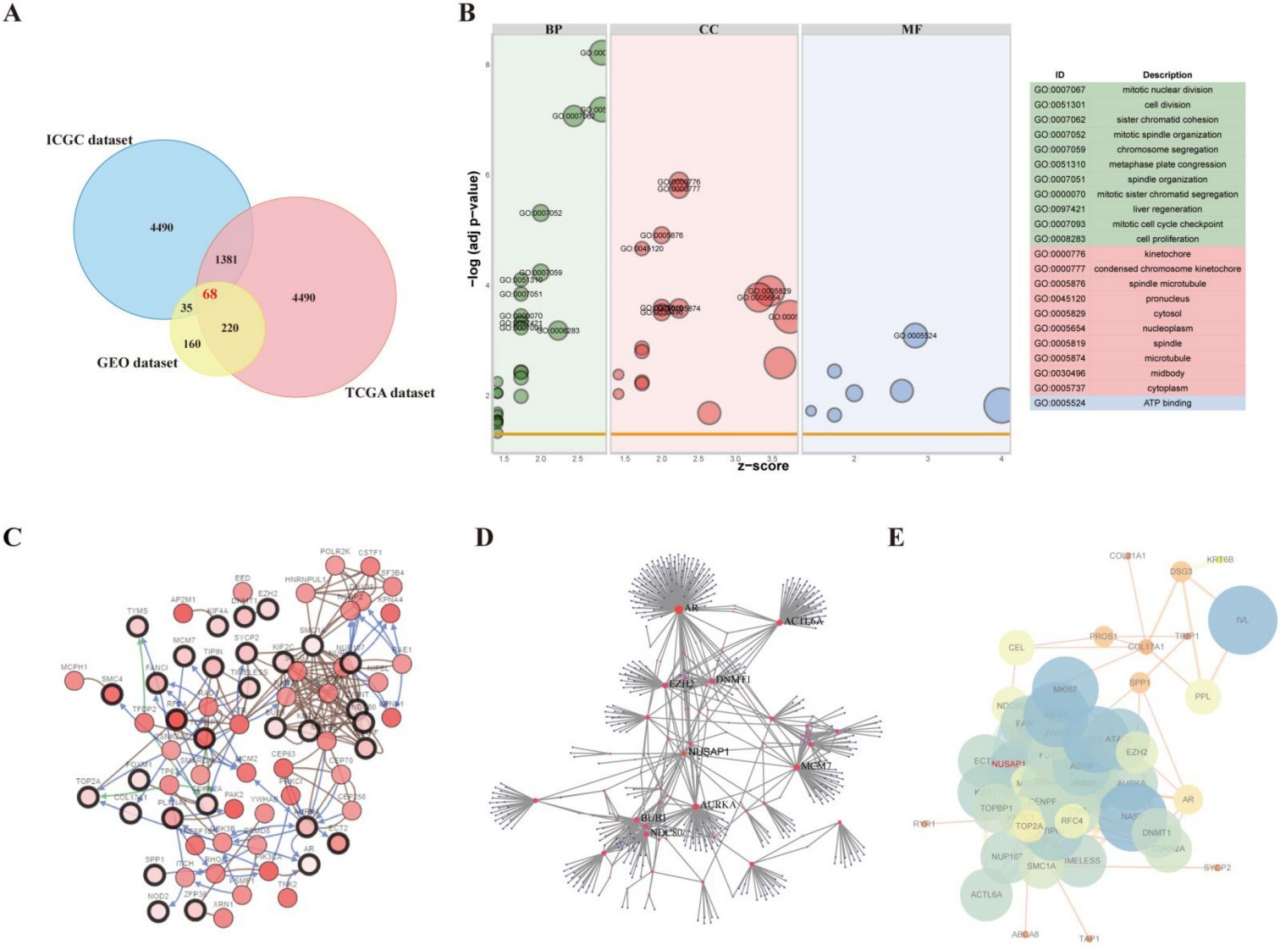
Identification of differentially expressed genes (DEGs) in cervical squamous cell carcinoma and endocervical adenocarcinoma (CESC). A. Venn diagram demonstrates the intersections of DEGs between GEO, TCGA, and ICGC data. B. Bubble plot of significantly enriched gene ontology (GO) terms for the DEGs. C. Co-expression analysis was performed using cBioportal. D. The protein-protein interaction (PPI) network of DEGs was generated using NetworkAnalyst. E. The PPI network of 68 DEGs is shown.

After standardization and removal of batch effects in the microarray results, 68 DEGs (1381 overlapping genes in ICGC and TCGA datasets; 35 overlapping genes in ICGC and GEO datasets; 220 overlapping genes in TCGA and GEO datasets) were identified (Figure 1A).

To explore the functions of these DEGs, GO terms including cellular component (CC), molecular function (MF), and biological process (BP) were analyzed by DAVID (Figure 1B). The DEGs were enriched in cell division, sister chromatid cohesion, mitotic nuclear division, mitotic sister chromatid segregation, mitotic spindle organization, and cell proliferation terms for BP. For CC, the DEGs were enriched in kinetochore, condensed chromosome kinetochore, cytoplasm, nucleus, chromosome (centromeric region), and nucleoplasm terms, and for MF, they were enriched in DNA binding, protein binding, and chromatin binding terms. The significantly enriched GO terms might facilitate the identification of potential biomarkers for CESC development.

### Construction of gene co-expression and protein-protein interaction networks

The network of DEGs and co-expressing genes, analyzed using the cBioPortal online platform, is shown in Figure 1C. The obtained DEG modules were analyzed using NetworkAnalyst platform and the PPI network was constructed (the confidence score was set at 900) (Figure 1D). The PPI network visualized using the NetworkAnalyzer plugin in Cytoscape software is shown in Figure 1E.

### GSEA analysis of CESC-related genes

**Figure 2:**
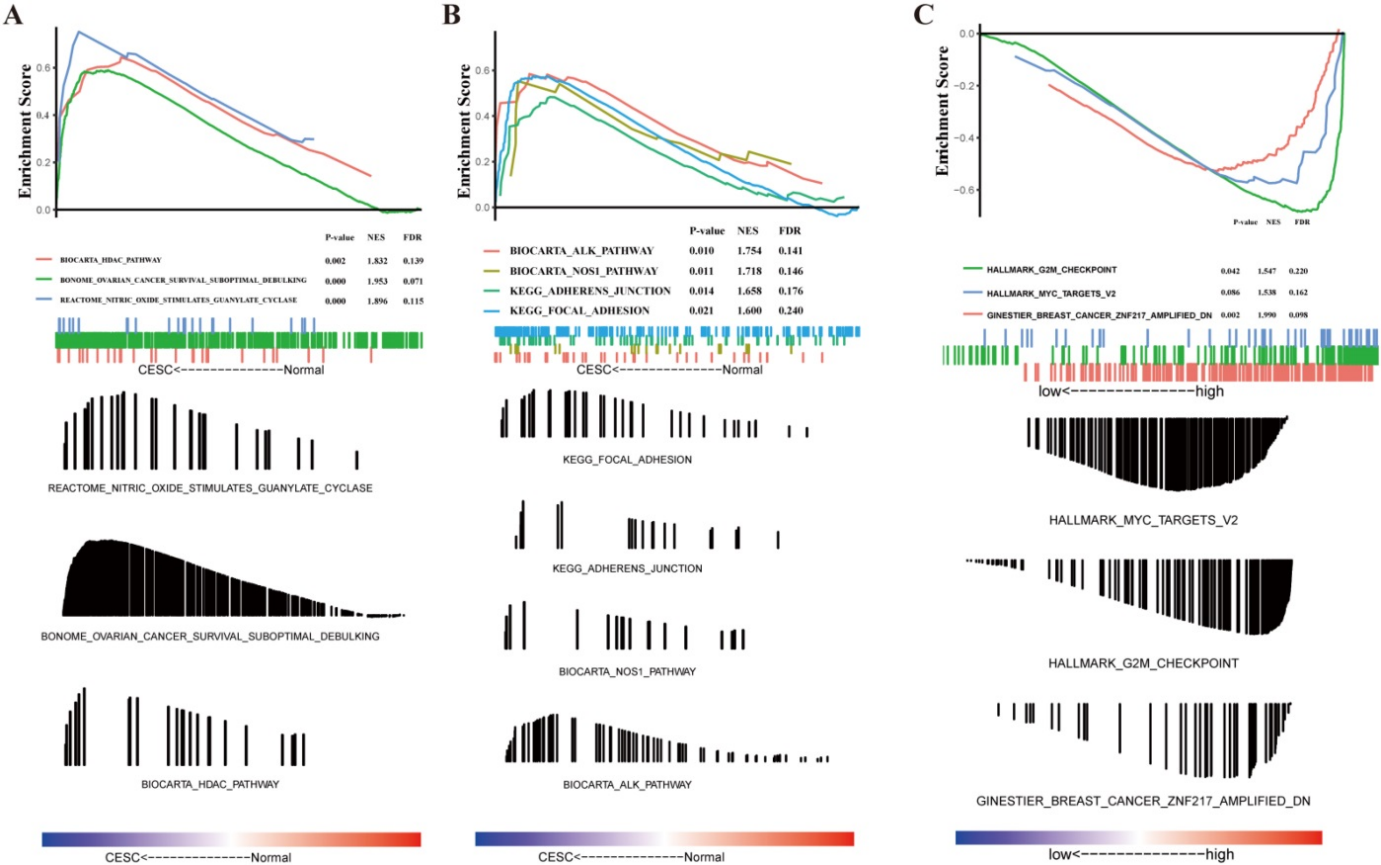
Gene set enrichment analysis (GSEA) indicating statistically significant enrichment (A-B) from cervical squamous cell carcinoma and endocervical adenocarcinoma (CESC) patients and a representative gene, NUSAP1 (C).

The molecular pathways that were significantly altered in CESC patient tissues compared to that in normal tissues were determined using the GSEA software. The GSEA of gene expression profiles was used to identify differentially enriched signaling pathways between patients with CESC and those that did not have CESC. These results indicate that CESC is predominantly associated with HDAC pathway, ovarian cancer suboptimal debulking, nitric oxide stimulated guanylate cyclase (Figure 2A), ALK pathway, NOS1 pathway, adherent junction, and focal adhesion (Figure 2B).

### Patient characteristics

As shown in Supplemental Table S1-S3, clinical information and gene expression data for 296 CESC cases were downloaded from TCGA database in October 2018. The median age of the diagnosed patients was 60. 93 (49.47%) patients were in stage I, 53 (28.19%) were in stage II, and 23 (12.23%) were in stage III. With respect to the CESC status of patients, 163 (68.78%) were tumor-free and 74 (31.22%) had tumor.

### Relationship between NUSAP1 expression and clinicopathological variables

**Figure 3:**
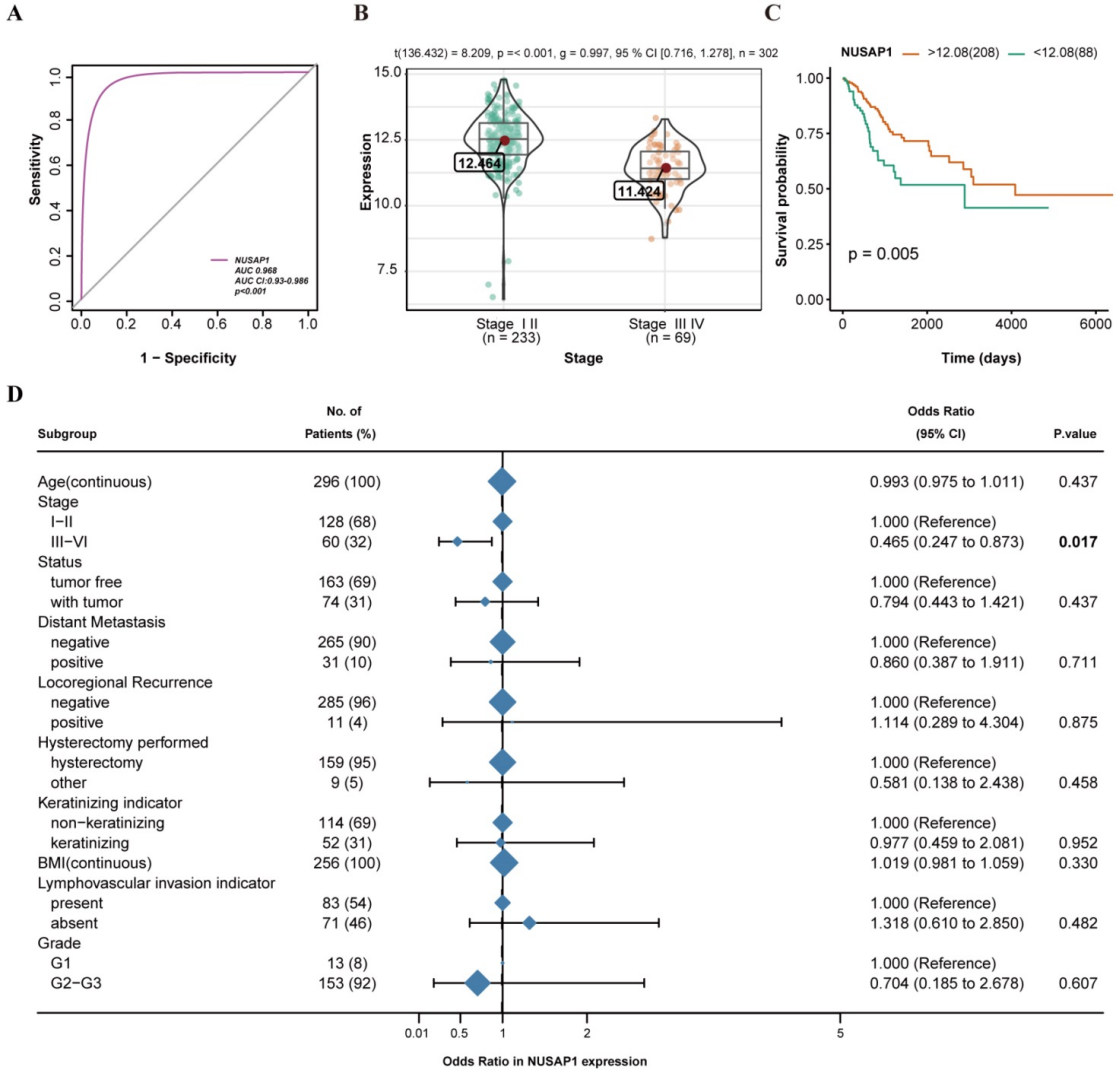
Association of NUSAP1 expression with clinicopathologic characteristics. A. ROC curve analysis of NUSAP1 expression, sorted by area under the curve (AUC), in cervical squamous cell carcinoma and endocervical adenocarcinoma (CESC) to examine the validity of NUSAP1 gene expression in discriminating tumor and non-tumor states of the CESC samples in an integrated analysis of GSE7803, GSE9750, and GSE63514 datasets. B. Association of NUSAP1 expression with the clinical stage. C. Effect of NUSAP1 expression on the overall survival of CESC patients in TCGA database. D. NUSAP1 expression related to clinicopathological characteristics (logistic regression).

As shown in Figure 1, NUSAP1 was listed as one of the DEGs in CESC. To identify the diagnostic value of NUSAP1 in CESC patients, ROC curve analysis was performed. The AUC value of the ROC curve of NUSAP1 was 0.968 (95% confidence interval [CI] = 0.930-0.986), suggesting the potential diagnostic roles of NUSAP1 in CESC (Figure 3A). A total of 296 CESC tissue samples, for which the NUSAP1 expression data and all patient characteristics were obtained from TCGA, were analyzed. The correlations between NUSAP1 expression and major clinicopathological factors were determined using logistic regression, as shown in Figure 3D. Reduced expression of NUSAP1 in CESC was significantly related to the advanced stage (OR = 0.465 for stages III-IV vs. stages I-II; P = 0.017) (Figure 3B). This indicates that CESCs with low NUSAP1 expression may progress to a more advanced stage than those with high NUSAP1 expression. Moreover, the effects of NUSAP1 on the overall survival of 296 CESC patients were analyzed using the Kaplan-Meier curve (P = 0.005) (Figure 3C). A cut-off value of 12.8 was set for assigning the patients to low or high groups for the best separation.

### Univariate and multivariate analysis

**Figure 4:**
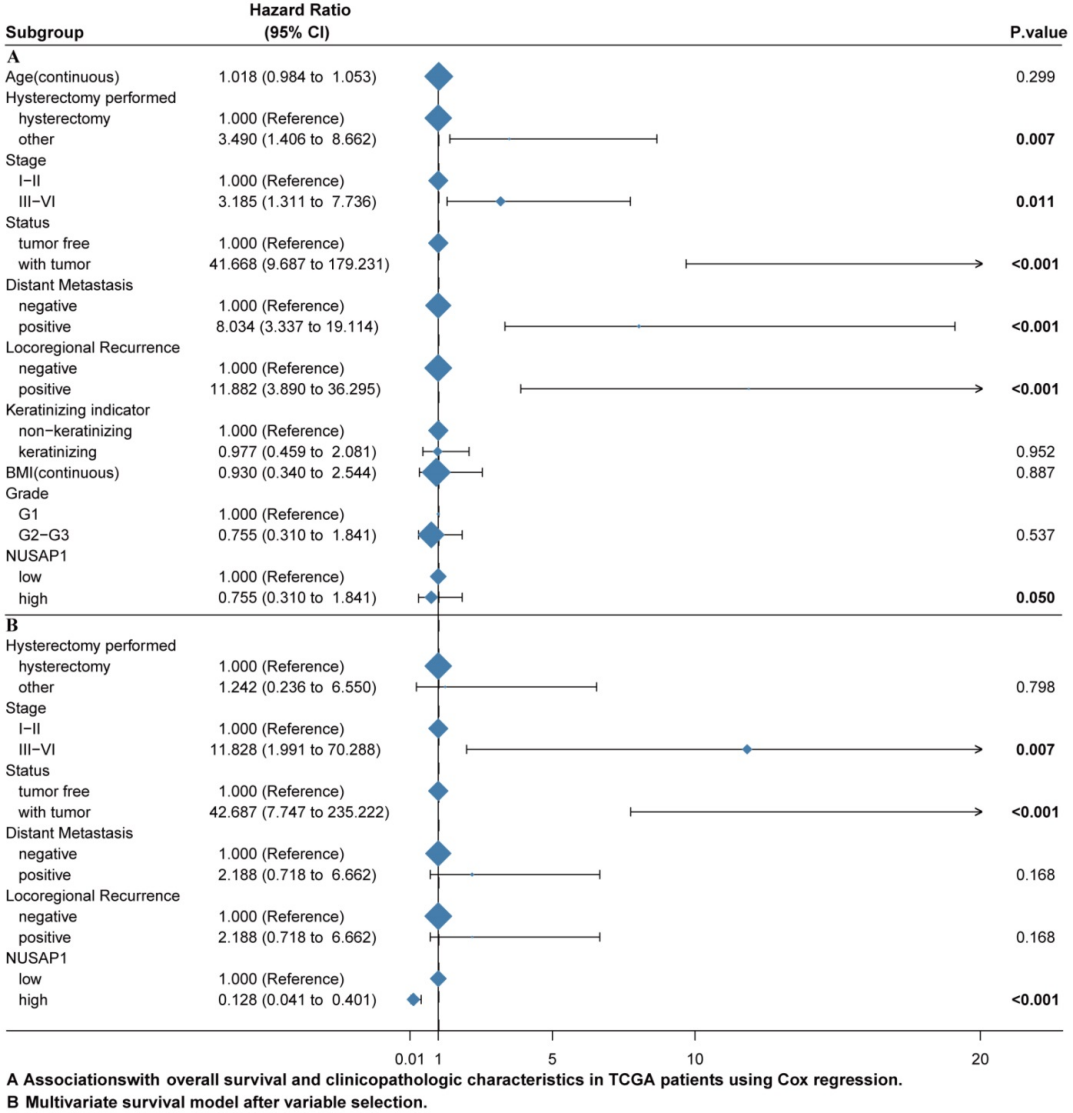
NUSAP1 expression related to prognostic factors. A. Cox regression analysis was performed to identify the factors that were associated with NUSAP1, affecting mortality in cervical squamous cell carcinoma and endocervical adenocarcinoma (CESC) patients and tumor recurrence in TCGA database. B. Multivariate survival model after selection of variables.

The univariate and multivariate Cox regression analyses were performed to investigate whether NUSAP1 is an independent predictor of poor survival in CESC patients after excluding the data of other patients with incomplete data. We included 138 patients with CESC for the Cox regression analysis. The univariate Cox regression analysis revealed that NUSAP1 was closely correlated with the overall survival (OS) (hazard ratio [HR] = 0.422, 95% CI = 0.178-1.001; P = 0.050). Moreover, other clinicopathological variables, including hysterectomy performed type (HR = 3.490, 95% CI = 1.406-8.662; P = 0.007), tumor stage (HR = 3.185, 95% CI = 1.311-7.736; P = 0.011), tumor status (HR = 41.668, 95% CI = 9.687-179.231; P < 0.001), distant metastasis (HR = 8.034, 95% CI = 3.337-19.114; P < 0.001), locoregional recurrence (HR = 11.882, 95% CI = 3.890-36.295; P < 0.001), were significantly related to OS (Figure 4A). In the multivariate Cox regression analysis, the tumor stage (HR = 11.828, 95% CI = 1.991-70.288; P = 0.007), tumor status (HR = 42.687, 95% CI = 7.747-235.222; P < 0.001), locoregional recurrence (HR = 10.782, 95% CI = 2.223-52.296; P = 0.003), and NUSAP1 (HR = 0.128, 95% CI = 0.041-0.401; P < 0.001) were significantly related to OS (Figure. 4B). All these data showed that NUSAP1 is an independent indicator for predicting a poor OS in CESC patients.

### Identification of NUSAP1-related signaling pathways using GSEA

To investigate the potential mechanisms that were activated in CESC, the GSEA of gene expression profiles was conducted to identify differentially enriched signaling pathways between CESC patients with high and low levels of NUSAP1 expression. We observed that G2M checkpoint, MYC target V2, and breast cancer ZNF217 were differentially enriched in the high NUSAP1 expression phenotype (Figure 2C).

### Expression of NUSAP1 in cancer cell lines and cancer tissues

**Figure 5:**
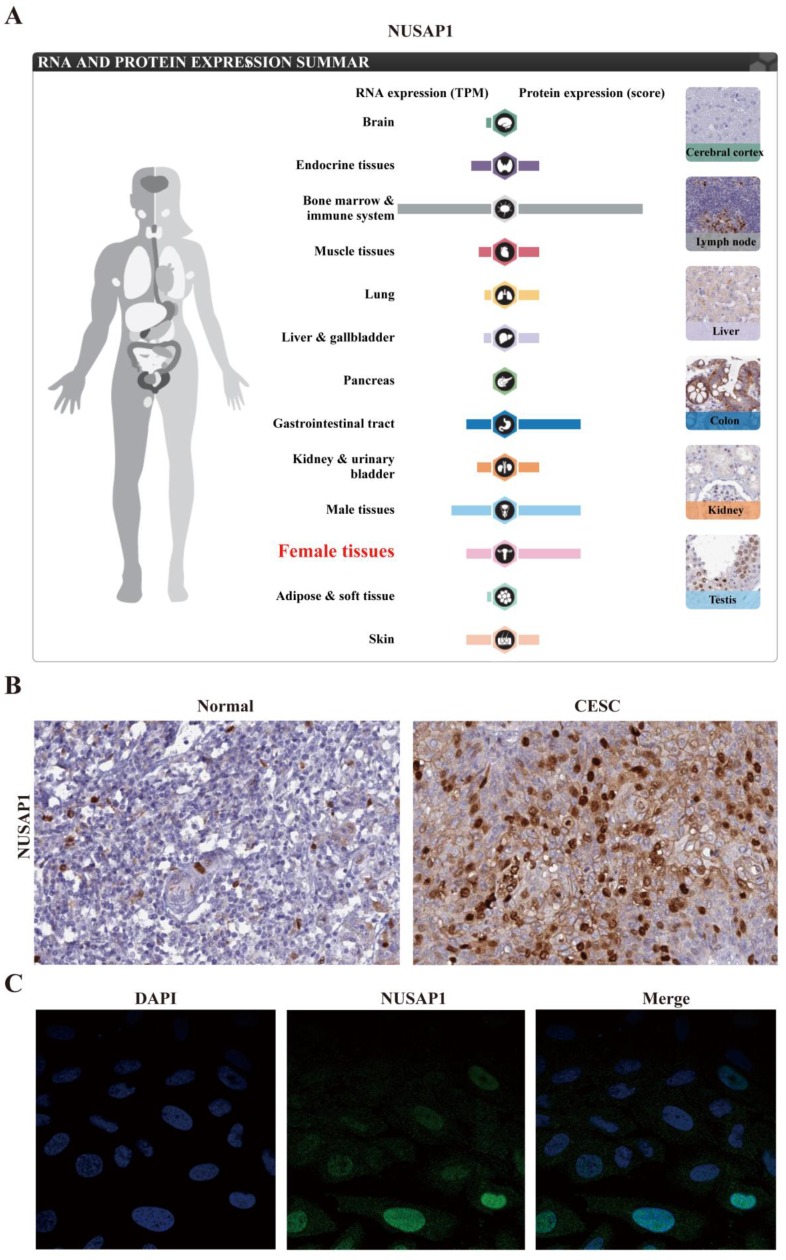
Expression of NUSAP1 at mRNA and protein levels in cervical cancer patients. A. Expression of NUSAP1 at mRNA and protein levels in different normal human tissues. B. Gene expression of NUSAP1 was assessed using immunohistochemistry in normal and cervical cancer tissues. C. Gene expression of NUSAP1 was identified using immunofluorescence in SiHa cell lines. All data are from The Human Protein Altas databases.

**Figure 6:**
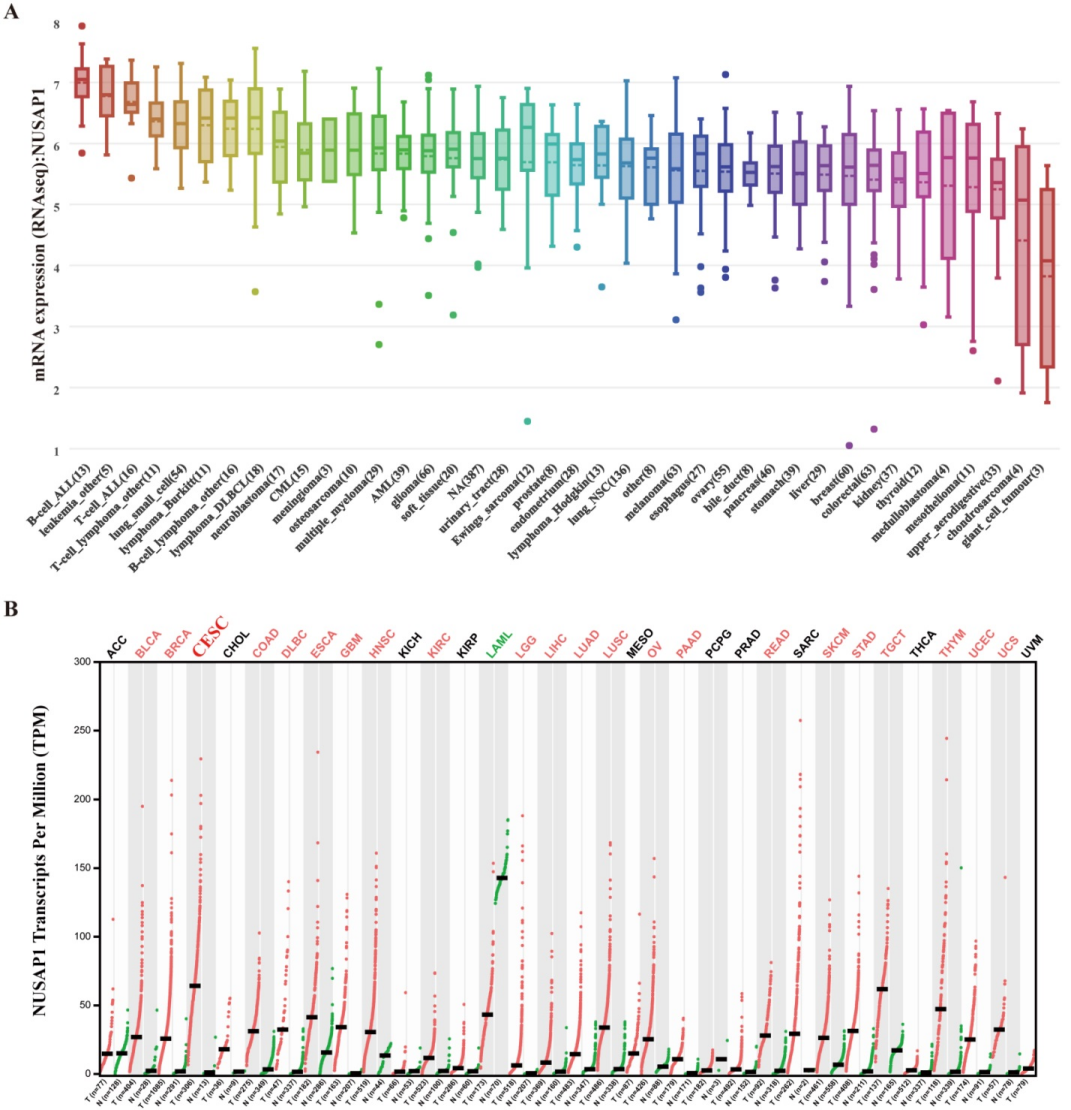
Expression of NUSAP1 at mRNA levels in different human tissues. A. NUSAP1 expression patterns in 1457 cell lines representing 40 distinct tumor types. B. Expression patterns of NUSAP1 in 33 cancer types and paired non-tumor samples.

The upregulation of NUSAP1 was found to be a common character in female and gastrointestinal tract tissues (Figure. 5A). The immunohistochemical analysis also demonstrated higher NUSAP1 levels in significant portions of CESC compared to that in normal tissues (Figure. 5B). As revealed by CCLE analysis, the NUSAP1 expression levels in B cell lines and leukemia cell lines were high. Immunocytochemical analysis to determine the expression pattern of NUSAP1 revealed that NUSAP1 fluorescence was partly distributed in the nucleus in SiHa cell lines (Figure 5C). Moreover, The expression of NUSAP1 mRNA in different cancer cell lines is shown in Figure 6A. Furthermore, the expression of NUSAP1 was high in 33 cancer types used for GEPIA, which computed TCGA data in the form of transcripts per million (Figure 6B). All these results indicate that the expression of NUSAP1 is significantly upregulated in CESC.

## Discussion

Emerging evidence suggests that CESC carries high risks of mortality and morbidity, which result from metastasis and recurrence, and the incidence to mortality ratio is nearly 50% 21. Genetic factors play a key role in tumorigenesis and cancer progression 22. However, the exact mechanism underlying CESC remains unknown. As of date, a number of oncogenes and cancer suppressor genes have been identified, some of which, including minichromosome maintenance complex component 2 (MCM2), DNA topoisomerase II alpha (TOP2A), and cyclin dependent kinase inhibitor 2A (CDKN2A), have been reported in CESC 23. Nevertheless, novel classes of biomarkers with high specificity, sensitivity, and efficiency are required for the diagnosis and prognosis of CESC. In this study, we explored the gene expression profile and pathological mechanism of CESC using microarray-based bioinformatic analysis. We identified a new gene, NUSAP1, which is closely related to apoptosis, proliferation, and metastasis, and there is little research on its mechanism in CESC, as yet. We comprehensively analyzed its expression and clinical relevance and explored its potential diagnostic and prognostic values in CESC.

NUSAP1 is a cell cycle-related protein that plays a key role in mitosis. It has been proved that NUSAP1 regulates apoptosis, proliferation, and metastasis 24. Accumulating evidence reveals that NUSAP1 may serve as a biomarker for carcinogenesis and cancer progression 25. The latest study demonstrates NUSAP1 has been shown to promote CESC via Wnt/ β-catenin signaling. And with RT-PCR and western blot to NUSAP1 is upregulated in cervical cancer 26. Besides in the present study, we found that NUSAP1 has a relatively high diagnostic performance with an AUC of 0.968 for CESC in an integrated analysis of GSE7803, GSE9750, and GSE63514 datasets. Similarly, NUSAP1 was identified as a marker for screening, with an AUC of 0.917, and it showed high sensitivity and specificity for diagnosis 8. Moreover, we found high expression of NUSAP1 at mRNA and protein levels in different CESC cell lines and tissues in CCLE and The Human Protein Atlas. Our results demonstrate that NUSAP1 is significantly overexpressed in CESC compared to that in normal cervical tissues. The mRNA and protein levels of NUSAP1 were reported to be significantly higher in colon tumor than in paired noncancerous adjacent tissues (P < 0.001, separately) 26. Using reverse transcription-polymerase chain reaction (RT-PCR) and western blot techniques, NUSAP1 was also reported to be overexpressed in tissue specimens and cell lines of renal cell carcinoma 27. Similar upregulation of NUSAP1 was also reported in prostate cancer cells 28 and hepatic carcinoma 29. In general, NUSAP1 is differentially expressed in tumor and adjacent normal tissues. However, further prospective investigations are warranted to establish the diagnostic accuracy of NUSAP1 in cervical cancer.

The obtained results reveal the diagnostic value of NUSAP1. However, the correlation between the expression of NUSAP1 and clinicopathological factors needs to be thoroughly investigated. A significant association between NUSAP1 expression and the FIGO stage was observed. The expression of NUSAP1 in CESC was higher in stage I and II patients than in stage III and IV patients. However, no significant association was observed between NUSAP1 expression and disease status, distant metastasis, tumor recurrence, uteri involvement, lymphovascular invasion, and histological grade. The study also found that the expression of NUSAP1 correlated with the tumor stage in renal cell carcinoma and colon cancer 26, 27. Conversely, one study found that overexpression of NUSAP1 was closely related with tumor size (P = 0.016), distant metastasis (P = 0.023), and Fuhrman grade (P < 0.001) 27. Furthermore, expression of NUSAP1 was significantly related to lymph node metastasis (P < 0.001), depth of invasion (P = 0.001), and histopathological grading (P < 0.001) 26. Hence, the correlation between NUSAP1 and clinicopathological factors needs to be further validated.

Previous studies have revealed that the dysfunction of NUSAP1 is associated with poor prognosis in multiple malignant tumors. Remarkably, overexpression of NUSAP1 (HR = 4.136, 95% CI = 1.956-8.747, P < 0.001) was identified as an independent prognostic indicator of disease-free survival in triple-negative breast cancer 30. Similarly, high expression of NUSAP1 was related to a shorter overall survival in patients with renal cell carcinoma (RCC) (P = 0.006) and colon cancer (P < 0.001) 26. We assessed the prognostic value of NUSAP1 in CESC patients. However, we found that CESC patients with lower NUSAP1 expression had poorer survival in selected datasets (HR: 0.52, 95% CI = 0.31-0.88; P = 0.52). Tumor progression is often accompanied by invasion and metastasis, which predict poor survival 31. To specifically address whether NUSAP1 is an indicator of good prognosis, it would be necessary to perform experiments at molecular and cellular levels to explore the mechanisms in which NUSAP1 is involved in patients with poor survival outcomes.

In the present study, GSEA was performed to investigate the potential signaling pathways of NUSAP1 in cervical cancer. Our results demonstrate that CESC patients with high NUSAP1 expression were enriched in the G2M checkpoint, MYC targets, and breast cancer ZNF217, which are closely related to the early onset of cancer and the risk of tumor progression and metastasis. The NUSAP1 overexpression is heavily implicated in microtubule bundling and cell cycle arrest at the G2/M checkpoint 32. Interestingly, MYC does not influence the NUSAP1 transcript levels in prostate cancer cell lines (LNCaP and PC-3) 28. It remains to be further examined whether overexpression of NUSAP1 affects the activation of MYC in CESC. Notably, the transcription factor zinc-finger protein 217 (ZNF217) is an oncogenic protein that is overexpressed in several tumors, including breast 33, ovarian 34, colon, and pancreatic cancers 35. The expression of ZNF217 is significantly enhanced in cervical cancer compared to that in normal cervical tissues 36. More studies are needed at the cellular and molecular levels to confirm whether dysfunctions of NUSAP1 regulate the pathways of ZNF217. Taken together, these findings demonstrate that NUSAP1 may act as a cancer suppressor in the development of CESC. It will be fascinating to unravel the molecular mechanism underlying the abnormal expression of NUSAP1 in CESC.

However, there are some notable limitations of the present study. First, the sample size was relatively low in the multivariate Cox regression analysis because of the lack of completed clinical data in TCGA; hence, the statistical power might be low. Moreover, the stratification analysis and interaction between the factors cannot be investigated further. In addition, the selective bias and recall bias were inevitable in the retrospective design. Therefore, further studies with a large sample size and prospective design are warranted to increase the statistical power and to achieve more meaningful outcomes. Second, despite the fact that microarray-based bioinformatic analysis is a powerful tool in efficient understanding of molecular mechanisms and for identifying potential biomarkers underlying CESC, further experimental validations of NUSAP1 are needed at molecular, cellular, and organismal levels.

In summary, integrated bioinformatic analyses of CESC datasets from TCGA, GEO, and ICGC databases revealed 68 DEGs, potential molecular mechanisms, and key pathways involved in CESC. Our results suggest that NUSAP1 may be a potential diagnostic and prognostic molecular marker for poor survival in CESC. However, large prospective studies are warranted to identify the prognostic value of NUSAP1 and further experimental validation should be performed to prove the biological role of NUSAP1 in CESC.

## Supplementary Material

Supplementary figures and tables.

## Abbreviations

AUC: area under curve
BP: biological process
CC: cellular component
CCLE: Cancer Cell Line Encyclopedia
CESC: Cervical squamous cell carcinoma and endocervical adenocarcinoma
DEGs: differentially expressed genes
FC: fold change
GEO: Gene Expression Omnibus
GO: gene ontology
HR: hazard ratio
GEPIA: Gene Expression Profiling Interactive Analysis
ICGC: international cancer genome consortium
MF: molecular function
NES: normalized enrichment score
NUSAP1: Nucleolar and spindle-associated protein 1
OS: overall survival
PPI: protein-protein interaction
TCGA: the Cancer Genome Atlas
ROC: Receiver operating characteristic
