# Supplementary material for: Decreased Expression of NUSAP1 Predicts Poor Overall Survival in Cervical Cancer
Source: J Cancer. 2020 Feb 21;11(10):2852–63. doi: 10.7150/jca.34640 (PMC7086256; doi:10.7150/jca.34640)

Supplemental Materials.

Supplemental Table S1

TCGA cervical cancer patient characteristics.

Clinical characteristics		Total (296)	%
Age (y)	≥ 60	57	19.26
	< 60	239	80.74
Clinical Stage	I	93	49.47
	II	53	28.19
	III	23	12.23
T Classification	T1	86	50.59
	T2	61	35.88
	T3	13	7.65
	T4	10	5.88
N Classification	N0	90	74.38
	N1	31	25.62
Hysterectomy Performed Type	Radical Hysterectomy	154	91.12
	Simple Hysterectomy	6	3.55
	Other	9	5.33
Keratinizing	Keratinizing squamous cell carcinoma	52	31.33
	Non-keratinizing squamous cell carcinoma	114	68.67
Lymphovascular Invasion	Absent	71	46.10
	Present	83	53.90
Measure of Response	Complete Response	83	74.77
	Partial Response	9	8.11
	Radiographic Progressive Disease	16	14.41
	Stable Disease	3	2.70
Neoplasm Histologic Grade	G1	13	7.78
	G2	82	49.10
	G3	71	42.51
	G4	1	0.60
Distant Metastasis	Positive	31	10.47
	Negative	265	89.53
Locoregional Recurrence	Positive	11	3.72
	Negative	285	96.28
Menopause Status	Peri(6-12 months since last menstrual period)	25	10.64
	Pre (<6 months since LMP AND no prior bilateral ovariectomy AND not on estrogen replacement)	127	54.04
	Post (prior bilateral ovariectomy OR >12 mo since LMP with no prior hysterectomy)	83	35.32
Status	With tumor	74	31.22
	Tumor free	163	68.78

Supplemental Table S2

TABLE S2. NUSAP1 expression* related to clinical pathological characteristics (logistic regression).

Clinical characteristic	Total(N)	Odds ratio in NUSAP1 expression	p-value
Age (continuous)	296	0.993(0.975-1.011)	0.437
Stage (III-VI vs. I-II)	188	0.465(0.247-0.873)	0.017
Status (with tumor vs. tumor free)	296	0.794(0.443-1.421)	0.437
Distant Metastasis (negative vs. positive)	296	0.860(0.387-1.911)	0.711
Locoregional Recurrence (negative vs. positive)	296	1.114(0.289-4.304)	0.875
Uteri involvement (absent vs. present)	118	1.524(0.407-5.710)	0.532
Hysterectomy performed (other vs. hysterectomy)	169	0.581(0.138-2.438)	0.458
Keratinizing indicator (non-keratinizing vs. keratinizing)	166	0.977(0.459-2.081)	0.952
BMI (continuous)	256	1.019(0.981-1.059)	0.330
Lymphovascular invasion indicator (absent vs. present)	154	1.318(0.610-2.850)	0.482
Grade (G2-G3 vs. G1)	167	0.704(0.185-2.678)	0.607

* Categorical dependent variable, less or greater than the median expression level.

Supplemental Table S3

TABLE 3. a. Relationship between overall survival and clinicopathologic characteristics in TCGA patients analyzed with Cox regression. b. Multivariate survival model after variable selection.

Clinicopathologic variable	HR (95% CI)	p-Value
a		
Age (continuous)	1.018(0.984-1.053)	0.299
Hysterectomy performed Type (other vs. hysterectomy)	3.490(1.406-8.662)	0.007
Stage (III IV vs. I II)	3.185(1.311-7.736)	0.011
Status (with tumor vs. tumor free)	41.668(9.687-179.231)	0.000
Distant Metastasis (negative vs. positive)	8.034(3.337-19.114)	0.000
Locoregional Recurrence (negative vs. positive)	11.882(3.890-36.295)	0.000
BMI (continuous)	0.930(0.340-2.544)	0.887
NUSAP1(low vs. high)	0.422(0.178-1.001)	0.050
Grade (G2 G3 vs. G1)	0.755(0.310-1.841)	0.537
b		
Hysterectomy performed Type (other vs. hysterectomy)	1.242(0.236-6.55)	0.798
Stage (III VI vs. I II)	11.828(1.991-70.288)	0.007
Status (with tumor vs. tumor free)	42.687(7.747-235.222)	0.000
Distant Metastasis (negative vs. positive)	2.188(0.718-6.662)	0.168
Locoregional Recurrence (negative vs. positive)	10.782(2.223-52.296)	0.003
NUSAP1 (high vs. low)	0.128(0.041-0.401)	0.000

Supplemental Figure S1

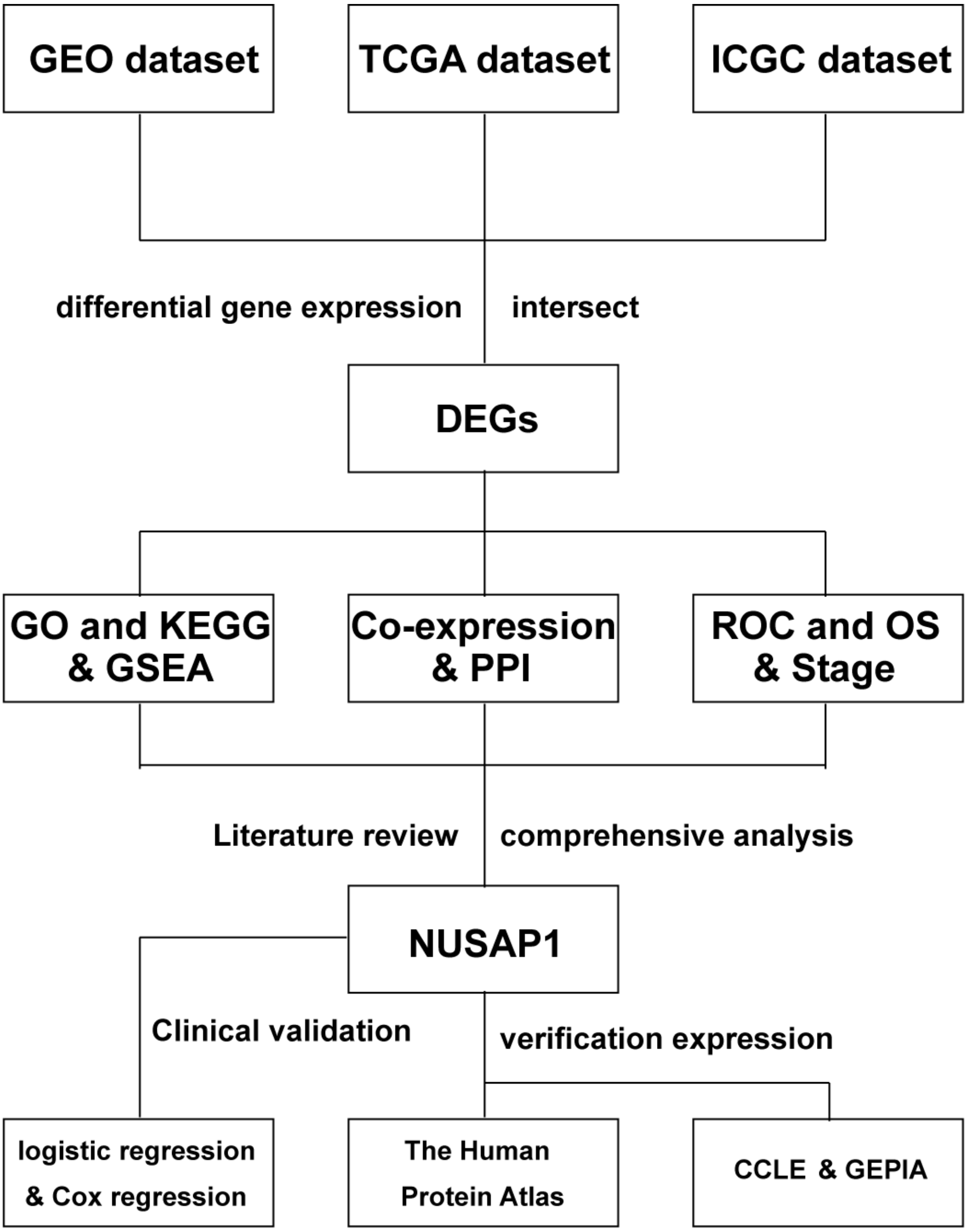

Supplemental Figure S2

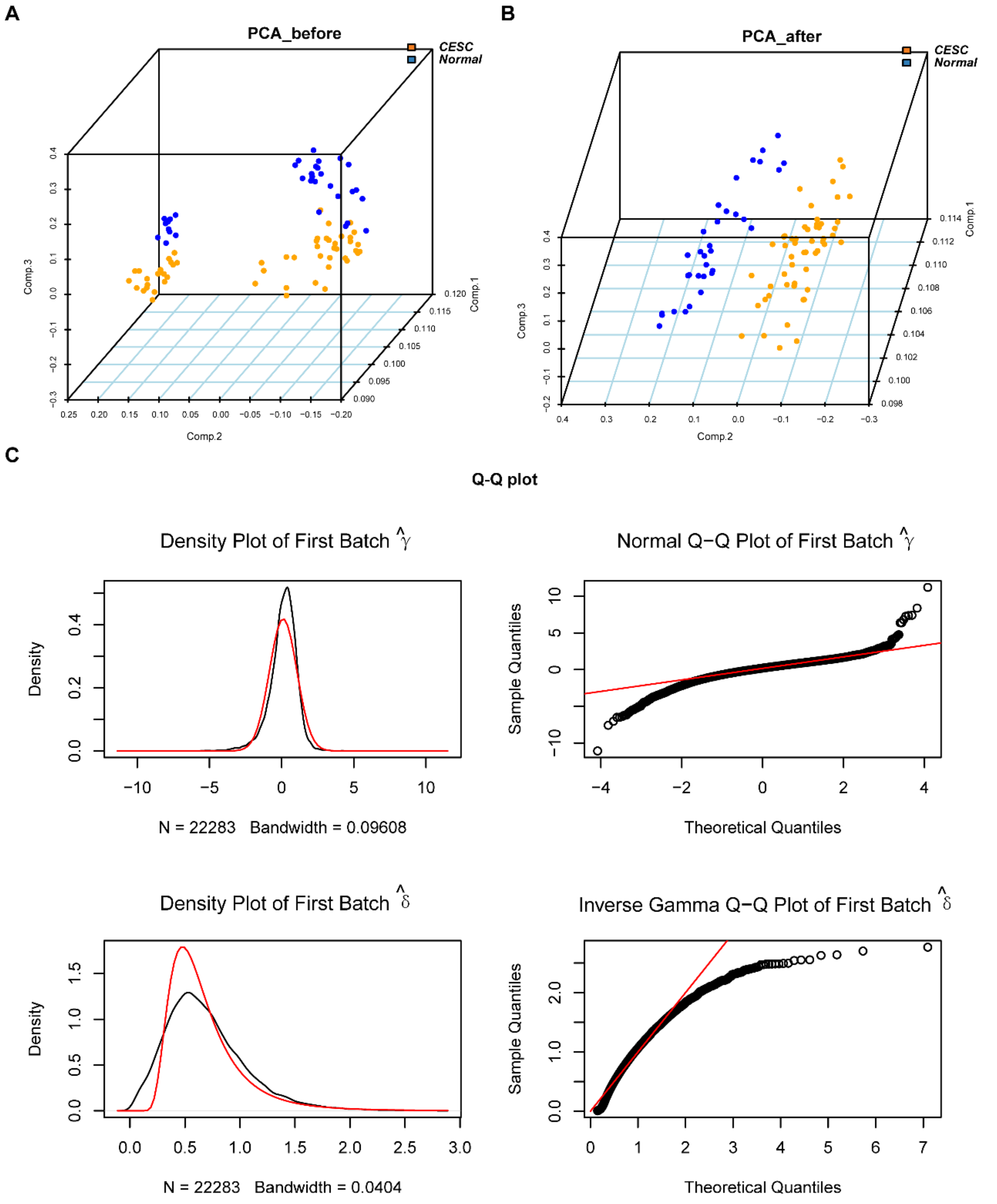

Supplement: Supplementary file 1 — Supplementary figures and tables. [file jcav11p2852s1.pdf]
